# Study of Brown-Sequard Theory

**Published:** 1889-10

**Authors:** Henry P. Loomis

**Affiliations:** New York


### AN EXPERIMENTAL STUDY OF THE BROWN-SEQUARD THEORY.

BY HENRY P. LOOMIS, M. D., NEW YORK.

The position which Dr. Brown-Sequard has held and still holds in the scientific world entitles any theory advanced by him to the respectful consideration of the profession. The recent one, relating to the use of injections of testicular liquid, was properly presented through reputable professional channels, and supported by experiments continued through a considerable period of time, and repeated and confirmed by other observers. Unfortunately, there has since been engrafted upon it much that has no shadow of foundation in any contention of the originator, and it has received a public and unprofessional notoriety which is not only to be regretted on general principles, but may possibly long prejudice us against any merit that it may actually possess. A brief review of his proposition is advisable.

The following is a fair statement of the physical effects of the treatment as applied by Dr. Brown-Sequard to himself:

From a condition of weakness, small appetite, and sleeplessness, he entered into one of comparative physical and mental strength. No record of the influence upon the appetite or capacity for sleep is given. He attributes these changes to an increase in the power of the nerve centres, and notes, upon a cessation of the injections, and after a period of four weeks, during which no marked change occurred, a gradual reversion to his former state. He denies that the beneficial effect is due to any personal idiosyncrasy, as experiments conducted by Dr. Variot independently of himself, upon five elderly men, produced similar results.

The conclusions he draws are:

1. That the spermatic fluid is potent to increase the strength of the human organism, presumably in old men, not by structural change, but by nutritive modification.

2. That the alterations in muscular structure not essentially allied to old age may disappear, and a consequent recovery of former power by the tissues may supervene; and,

Finally, that the subject is well worthy further experimental investigation.

In further definition of his position, Dr. Brown-Sequard specifically affirms his continued belief in the fatality and irreversibility of those nutritive actions which produce the changes of advancing age, and acknowledges that the facts presented are insufficient to solve the question of possible structural change in muscles, nerves and nerve-centres.

I can see nothing in this to support the accusation that he claims to have discovered the source of the perpetual youth, but rather a flat denial of any such contention, as though, in making his affirmation, he recognized the danger of misrepresentation and made an effort to provide against it. What he does claim is the discovery of certain physical changes wrought by the injection of the mixture; that it was apparently beneficial in its action with possibilities of becoming more so, and that the phenomena were important enough to invite careful study and analysis. At all events, my experiments have been made with such interpretation of the proposition, and the deductions given below are the present result.

Following Dr. Brown-Sequard's instruction, and always observing strict antiseptic precautions, I crushed in a mortar a small portion of the tissue of the testicle of a two-year-old ram, mingled with an equal quantity by bulk of distilled water.

The resultant mixture, after infiltration, disclosed under the microscope albuminous granules and crystals of spermine that are formed of a phosphate of an organic base, but no bacteria or spermatozoa. Thirty minims constituted a dose, and fresh material was prepared on each occasion. Not more than an hour was allowed to elapse between the death of the animal and the operation.

Within the last three weeks I have injected fluid of this character into a number of men, with the following results:

Class 1. includes only such as were patients in Bellevue Hospital, who had no knowledge or suspicion of the character of the liquid, and took no medicine during the period of experimentation.

Case 1. Charles G———, aged fifty-six, shoemaker, had been cured, but his asthmatic attacks still continued, coming on generally in the early evening. The patient was given four injections of thirty minims each of the fluid, at intervals of two days. A short time after the first injection he said "he felt as if he had taken a big dose of morphine;" that night he was not troubled with his asthma, for the first time in over a month. During the following day he felt much better, stronger, and with increased vitality. This improvement continued and increased up to the time of the fourth injection. After the fourth injection, although the patient complained of no pain at the seat of puncture, still for the twenty-four hours following the injection his general condition seemed to be much worse, his hands trembled, he complained of a burning sensation, and appeared as one who had suffered a severe nervous shock. At the expiration of the twenty-four hours his condition became substantially as it was preceding the last injection. The return to this condition took place about a week ago, since which time no injections have been administered. The seemingly beneficial effects have gradually worn off, accompanied by a slight return of his asthmatic attacks and loss of strength.

During the time of experimentation no change was noticed in his pulse, which ranged between 62 and 70. His respirations at the time of the first experiment were 24, after the first injection they remained about 20. The dynamometer moved by the flexors of the right forearm registered 57 as against 50 prior to the injections.

*Comments.*—During the period of experimentation the patient's asthma improved, his respirations became less frequent, his strength increased, and his general condition was better. The improvement in the patient has not been permanent.

Case 2. William D———, aged sixty-two, a tailor, sufferer from chronic diarrhœa, general debility and senility, while not actually on the sick list at the time of experimentation, had been in no particular materially benefitted by treatment. He received four injections at intervals of three days. The day following the first injection he acknowledged that he "felt better," but attributed his condition to an improvement in his diarrhœa. After the second and third injections he affirmed that he "felt much better, livelier, and clearer in his head;" "that he was much better and able to go about his work."

His pulse during the period of experimentation remained at 64, respiration at 20, and strength, as registered by the dynamometer, at 60.

*Comments.* The patient' statements of his improved condition were fully within the apparent facts.

Case 3. Matthew M———, aged seventy-seven, a sufferer from senility, was under special treatment for diarrhœa, which had lasted for about three weeks and had been controlled by appropriate remedies. He received three injections at intervals of two days, the first being given, as in other cases, at about 5 p. m. By the patient's own statement, his improvement began to be noticeable on the morning following the first injection. His words in describing his condition were, that he "felt much livelier and better," and the testimony of the attendant physicians and nurses was to the same effect. The estimate of the improvement in this case is much more emphatic than the others. The pulse was variable both before and during the period of experimentation, marked increase, however, being observed during the twelve hours immediately following the injection. Respiration, which prior to the injection had averaged 16, then rose to 20. The registration on the dynamometer showed an increase from 38 to 42.

*Comments.*—I regarded this case as the one best calculated to test the efficacy of the fluid. The improvement, while in no way sudden or extraordinary, has been steady and decided. As insufficient time has elapsed since the last injection, it is premature to estimate the permanency of the improvement.

Case 4. G. D———, aged fifty-two, a sufferer from chronic rheumatism, received a single injection without general or specific result.

Case 5. John C———, aged fifty-seven, seaman, had been suffering for three weeks from the exacerbation of a chronic rheumatism. He received two injections of 30 minims each with an interval of forty-eight hours. Not only was no improvement noticed, but rather an increase of symptoms.

Class 2. embraces, with one exception, private cases. All men of intelligence and education, and were familiar with the theory and method of treatment.

Cas 6. L. K———, aged forty-eight, with a neurotic family history, suffered from an entire loss of sexual appetite, due no doubt, to excesses in early life. He complained of loss of memory, insomnia, and despondency. He had no erection since last March. Two injections of 30 minims each, with an interval of three days, were given him without result.

Case 7. George D———, aged thirty-five, was a sufferer from no organic difficulty, but was a typical nervous case, and had consulted many physicians with no improvement, as he thought. He was confident this remedy would effect his permanent cure. He received four injections at intervals of two days, with a marked increase of nervousness. He seemed to be made worse by the treatment.

Case 8. Doctor M———, aged fifty-seven, suffered for the past five years from constant anæmic headache; with this exception he was perfectly well. As he had obtained no relief from medication, he requested me to give him an injection. He derived no appreciable benefit from the injection, and his headache was not mitigated.

Case 9. J. A. R———, aged thirty-six, a sufferer from what his physician diagnosed as chronic muscular rheumatism, obtained no improvement from two injections.

Case 10. X. Y———, aged forty-seven, had suffered for five years from a peculiar nervous disease which had been diagnosed, by some specialists in Europe and this country, as locomotor ataxia. As this gentleman has education and exceptional ability, and is free from all prejudice in regard to the treatment, I give his own description of the effect of the first injection, which was administered at 6 p. m. "I noticed no sensation," he said, "until 7:15 the same evening. Then a comfortable feeling came over me, as if I had taken a glass of champagne; in a word, I felt rested. On retiring, at nine o'clock, I noticed some soreness in my leg, in the region of the puncture. From this time until 2 a. m. I suffered from most peculiar sensations. I was very restless and nervous; my body felt as if it were burning up, my mouth was parched, and my face flushed. My wife, thinking I must have a high fever, took my temperature, but found it normal. At this time I was wide awake and my mind active. Most of the time I was unable to control the shaking of my hands. I slept from two o'clock until eight, when I awoke somewhat refreshed, and found that the soreness had left my leg. All the forenoon I felt sleepy, but by atfernoon I began to improve, and by evening I felt better than I had for months, stronger, and with more ambition. This effect has continued up to the present time (two days).

This patient is now under treatment, and has had four injections, but I can see no improvement in his disease. His gain, if any, is purely subjective.

I have received a number of reports of cases from physicians to whom I have furnished the fluid. Some of them state that there has been a wonderful improvement in their subjects. As, however, these cases have not fallen under my personal observation, I refrain from noting them. My observations upon the cases are as follows:

I. *a.* I can see no reason to anticipate danger of septicæmia from the use of the fluid prepared under proper antiseptic precautions, provided the material used be absolutely fresh and free from all trace of disease. My attention was called to the necessity for the closest scrutiny in this last particular, by having discovered, in specimens taken from an apparently healthy ram, a solitary tubercle in which were demonstrated tubercle bacilli. In none of the cases have I seen any bad results, and only in a few has there been a moderate amount of pain at the point of injection, lasting from six to eight hours.

*b.* I can explain the singular nervous affection apparent in certain of the cases only on the theory that upon the nerve-centres the mixture exerts some powerful but as yet unexplained influence, which, even if its use be eventually proved beneficial in some cases, must render its employment in others a matter of caution. It is far from safe to say and proceed upon the belief that "if it does no good it can do no harm."

II. *a.* I seem to see in almost all the cases of *old men* subjected to the experiment an increase in strength and vitality which certainly persists for several days. I have noticed nothing in the least resembling the secondary depression which so commonly follows the use of ordinary stimulants.

*b.* When used in cases of actual disease no modification of pathological conditions or processes has been recognizable.

I therefore conclude:

1. That the injection of this testicular mixture does, as claimed, produce "nutritive modification" in the tissues of elderly men, due probably to the stimulation of the nerve-centres.

2. As far as my own experiments are concerned, sufficient time has not yet elapsed to justify an affirmation or denial of the correctness of Dr. Brown-Sequard's second conclusion.

3. There is in the theory sufficient ground for further experimentation.—*Medical Record.*

